# Salt Tolerance Mechanism of the Rhizosphere Bacterium JZ-GX1 and Its Effects on Tomato Seed Germination and Seedling Growth

**DOI:** 10.3389/fmicb.2021.657238

**Published:** 2021-06-08

**Authors:** Pu-Sheng Li, Wei-Liang Kong, Xiao-Qin Wu

**Affiliations:** ^1^Co-Innovation Center for Sustainable Forestry in Southern China, College of Forestry, Nanjing Forestry University, Nanjing, China; ^2^Jiangsu Key Laboratory for Prevention and Management of Invasive Species, Nanjing Forestry University, Nanjing, China

**Keywords:** *Rahnella aquatilis*, salt tolerance mechanism, compatible solute, growth-promoting function, plant growth-promoting rhizobacteria

## Abstract

Salinity is one of the strongest abiotic factors in nature and has harmful effects on plants and microorganisms. In recent years, the degree of soil salinization has become an increasingly serious problem, and the use of plant growth-promoting rhizobacteria has become an option to improve the stress resistance of plants. In the present study, the salt tolerance mechanism of the rhizosphere bacterium *Rahnella aquatilis* JZ-GX1 was investigated through scanning electron microscopy observations and analysis of growth characteristics, compatible solutes, ion distribution and gene expression. In addition, the effect of JZ-GX1 on plant germination and seedling growth was preliminarily assessed through germination experiments. *R. aquatilis* JZ-GX1 was tolerant to 0–9% NaCl and grew well at 3%. Strain JZ-GX1 promotes salt tolerance by stimulating the production of exopolysaccharides, and can secrete 60.6983 mg/L of exopolysaccharides under the high salt concentration of 9%. Furthermore, the accumulation of the compatible solute trehalose in cells as the NaCl concentration increased was shown to be the primary mechanism of resistance to high salt concentrations in JZ-GX1. Strain JZ-GX1 could still produce indole-3-acetic acid (IAA) and siderophores and dissolve inorganic phosphorus under salt stress, characteristics that promote the ability of plants to resist salt stress. When the salt concentration was 100 mmol/L, strain JZ-GX1 significantly improved the germination rate, germination potential, fresh weight, primary root length and stem length of tomato seeds by 10.52, 125.56, 50.00, 218.18, and 144.64%, respectively. Therefore, *R. aquatilis* JZ-GX1 is a moderately halophilic bacterium with good growth-promoting function that has potential for future development as a microbial agent and use in saline-alkali land resources.

## Introduction

Soil salinization is a worldwide soil degradation problem that not only causes damage to resources but also harms environmental and economic development (Zhang et al., 2010). Therefore, the remediation, development and use of saline-alkali soil are of great significance. Some microorganisms can survive in adverse conditions, among which halophilic bacteria have attracted a great deal of attention due to their great potential applications. Therefore, elucidation of the mechanisms by which halophilic bacteria adapt to salt stress is of great significance in the future development and use of saline-alkali soil.

Microorganisms living under salt stress require specific adaptation mechanisms, and halophilic bacteria have been shown to use different strategies to deal with excessive salt in the external environment (Roy and Colin, 2002). The first strategy is the accumulation of potassium/chloride ions to maintain the internal osmotic balance of cells under high salt conditions (Hnelt and Volker, 2013), and adaptation that is common in extremely anaerobic halophilic bacteria (Oren, 2004). However, most halotolerant and moderately halophilic bacteria use a second strategy: the accumulation of compatible solutes. These complex solutes (including amino acids, carbohydrates and their derivatives, sugars and polyols) can be absorbed from the medium or synthesized by organisms themselves (Shivanand and Mugeraya, 2011). In cells, these solutes accumulate at high concentrations to maintain the balance between intracellular and extracellular osmotic pressure (Sabine et al., 2018), which is the most widely used salt tolerance strategy in bacteria, eukaryotes and some methanogenic bacteria (Lai and Gunsalus, 1992; Gupta et al., 2015). Studies have shown that betaine plays an important role in the maintenance of cell osmotic pressure. Betaine has the advantages of having no electrostatic charge and high solubility and does not affect many enzymes or other biological macromolecules, even at high concentrations (Yu et al., 2017). Betaine is the most common osmotic protective agent in prokaryotes (Farooq et al., 2015), and *Rhodococcus* sp. W2 has been reported to use intracellular betaine to resist high-salt environments (Shen et al., 2013). Trehalose is also a well-known osmotic protective agent (Jha et al., 2011). Under high-salt stress, the halophilic fungus *Aspergillus montevidensis* ZYD4 can alleviate the effects of salt stress by regulating the changes in cellular trehalose contents (Ding et al., 2019). In addition, many bacteria produce extracellular polysaccharides (EPSs) as a growth strategy that enables their survival under adverse conditions (Iwabuchi et al., 2003; Sivan et al., 2006; Urai et al., 2006; Perry et al., 2007). EPSs form a layer around cells to reduce cell damage under abiotic stress conditions, and the production of EPSs is essential for adhesion of other organisms, biofilm formation and nutrient absorption (Sutherland, 2001; Nichols et al., 2005).

In recent years, the biological engineering plants that are fully tolerant of saline-alkali environments has become a new research direction at home and abroad with the goal of improving plant yields in saline-alkali soil. At present, the use of plant growth-promoting rhizobacteria (PGPR) has been emphasized as an effective means improving soils with high-salt concentrations and promoting plant growth (Walia et al., 2005; Bashan, 2012). Studies on plant seed germination have shown that at a soil salt content of 0.2–0.3%, seedlings typically exhibit difficultly in emerging, while in soil with a salt content of 0.4–0.5%, seeds cannot unearth, and germination is difficult at soil salt contents greater than 0.65% (Liu et al., 1993). Mo et al. (2006) and other researchers have shown that *Arthrobacter* sp. Rs15-4 can improve the germination rate of cotton seeds and promote the growth of cotton seedlings under salt stress. In addition, many PGPRs have been shown to promote plant growth through a variety of other mechanisms. For example, indole-3-acetic acid (IAA) produced by PGPRs is an important plant hormone that controls many physiological processes (Spaepen et al., 2007; Leveau and Gerards, 2008). *Bacillus subtilis* pk5-26 can grow under severe salt stress and has a remarkable growth-promoting effect on *Arabidopsis thaliana*, as it is capable of dissolving phosphorus and secreting siderophores (Bokhari et al., 2019).

Preliminary studies conducted in our laboratory resulted in the isolation of the rhizosphere growth-promoting bacterium *Rahnella aquatilis* JZ-GX1 from the rhizosphere soil of 28-year-old Masson pine in Nanning, Guangxi. Strain JZ-GX1 exhibited high phytase activity, and its ability to produce IAA was shown to have significant growth-promoting effects on pine and poplar (Li and Wu, 2014). To date, research on *Rahnella* sp. has primarily focused on the biocontrol of plant diseases, the environmental remediation of selenium pollution, plant growth promotion, etc., while few studies have investigated the mechanisms associated with its salt tolerance. While PGPR aid in the ability of plants to resist stress, they must also deal with osmotic pressure changes. Thus, an in-depth understanding of the relevant mechanisms by which PGPRs cope with such stress is important for promoting PGPR-mediated strategies to improve plant abiotic stress. Thus, the goal of the present study was to elucidate the salt tolerance mechanism of *R. aquatilis* JZ-GX1 by investigating its growth characteristics under salt stress, accumulation of compatible solutes, ion distribution and expression of salt tolerance genes and to investigate the effect of this strain on tomato germination under salt stress. The results lay a foundation for expanding the application of this strain and promoting plant growth through the use of PGPRs to improve the ecological environment of salinized soil.

## Materials and Methods

### Tested Strains and Culture Medium

*R. aquatilis* JZ-GX1 is a plant growth-promoting bacterium isolated from the rhizosphere soil of 28-year-old *P. massoniana* in Nanning, Guangxi, and is stored in the Culture Preservation Center of China (CCTCC, No: M2012439). After activation, strain JZ-GX1 was cultured overnight in LB liquid medium at 28°C (Kong et al., 2020). LB solid medium was prepared with 10 g of peptone, 5 g of yeast powder, 10 g of NaCl, and 15–20 g of agar (pH 7.2), and the NaCl concentration of the medium was set to 0, 3, 6, and 9% (0, 0.51, 1.02, and 1.53 mol/L, respectively). The activated strains were inoculated into the prepared media and cultured to the logarithmic phase at 28°C with shaking 200 r/min.

### Determination of the Growth of *R. aquatilis* JZ-GX1 Under Salt Stress

Strain JZ-GX1 was inoculated on plates with different salt concentrations, and its growth on was observed after 24–48 h. Then, 50 μL of an activated bacterial solution was inoculated into test tubes containing 4,950 μL of LB medium with different salt concentrations and shaken. Subsequently, the culture broth was pipetted into each well of a 96-well plate and cultured in a microplate reader (Bioscreen C, FP-110-C, Finland) at 28°C to determine the growth curves, with measurements automatically collected every 2 h for 48 h. The activated strain was inoculated (1%) in LB medium with different salt concentrations, and after cultivating for 24, 48, and 72 h, the cells were collected by centrifugation and dried to a constant weight to determine the dry weight.

### Morphological Observation of Strain JZ-GX1 in the Presence of Different Salt Concentrations

Scanning electron microscopy observations of the tested strain JZ-GX1 were obtained as described by Castelijn et al. (2012). For sample preparation, the cells were first fixed with glutaraldehyde, washed with 0.2 M phosphate buffer (pH 7.4) and dehydrated with gradient ethanol. The dehydrated sample was dried with a CO_2_ critical point desiccator (liquid CO_2_, EMITECH K850), after which the dried sample was divided into fragments of suitable length, glued to the table and sprayed with gold (HITACHE-1010). Subsequently, the morphology of strain JZ-GX1 cells in the presence of different salt concentrations was observed with a scanning electron microscope (QUANTA200, FEI, United States).

### Determination of Extracellular Polysaccharides Produced by Strain JZ-GX1 in the Presence of Different Salt Concentrations

The EPS content in the culture medium was determined using the phenol-sulfuric acid method (Wang, 2015).

### Determination of Compatible Solutes Accumulated by Strain JZ-GX1 in the Presence of Different Salt Concentrations

LB media with different salinities (0, 3, 6, and 9%) was prepared. The bacteria were inoculated (1%) and cultured on a shaking table (28°C, 180 rpm) for 1, 2, 3, and 4 days. Subsequently, 500 μL of bacterial suspension was collected and centrifuged at 10,000 rpm for 3 min, and the supernatant was discarded.

Samples were prepared to assess the proline content of cells using a ratio of bacteria (10^4^cfu/mL) to extract volume (mL) of 500:1 (ultrasonic power 200 w, 3 s, interval 10 s, repeated 30 times). Thee samples were extracted in a 95°C water bath for 10 min. Then, after cooling, the samples were centrifuged at 25°C for 10 min at 10,000 rpm, and the supernatant was reserved. Samples were prepared to assess the trehalose content of cells by lysing bacteria with an ultrasonicator at a ratio of bacteria (10^4^ cfu/ml) to extract volume (mL) of 500:1 (ultrasonic power 200 w, 3 s, interval 10 s, repeated 30 times). Then, after incubating at room temperature for 45 min, the samples were shaken 3–5 times, cooled, centrifuged at 8,000 rpm at 25°C for 10 min, and the supernatant was reserved. Samples were prepared to assess the betaine contents of cells by continuously shaking the samples with 1.2 mL of water for 60 min prior to extraction for 30 s. Subsequently, 400 μL of extract was added, mixed thoroughly, and centrifuged at 10,000 rpm at 25°C for 10 min to obtain the supernatant.

Three replicates of each treatment were evaluated, and the proline, betaine and trehalose contents accumulated by strain JZ-GX1 in the presence of different salt concentrations were determined using a commercial kit (Suzhou Keming Biotechnology Co., Ltd.).

### Determination of the Na^+^ and K^+^ Concentrations of Strain JZ-GX1 in the Presence of Different Salt Concentrations

Na^+^ and K^+^ were extracted according to Nagata’s method, and the concentrations of Na^+^ and K^+^ were determined by flame photometry (Nagata et al., 2005).

### Differential Expression of Salt Tolerance-Related Genes in Strain JZ-GX1 in the Presence of Different Salt Concentrations

For quantitative RT-PCR, strain JZ-GX1 was cultured in LB medium for 72 h with shaking, after which RNA was extracted from cells using the TRIzol method. cDNA samples were prepared using HiScript II Q Select RT SuperMix for qPCR (China). The expression of salt tolerance-related genes was determined by qRT-PCR with an ABI 7500 instrument (Applied Biosystems, United States), and atpD was used as an internal control (Li et al., 2019). Three genes related to salt tolerance were selected from the genome of *R. aquatilis* JZ-GX1 (unpublished) for analysis (Table 1). The relative gene expression changes were calculated with the 2^−ΔΔCT^ method. The RT-PCR assay consisted of three independent experiments, with three replicates performed for each experiment.

**TABLE 1:** Primers used in the RT-qPCR analysis.

Gene name	Gene functions	Primers
nhaB	Na^+^/H^+^ antiporter NhaB	CGCTATTACTTCTCCAATCC ATCCACAATCTGCTCGTA
betB	Betaine-aldehyde dehydrogenase	ATGCATCACCCGCAGTTGTA TGAACGCTTTACCGCTCAGT
lgbT	L-Proline/glycine/betaine transporter	ATGAGCACTTCAACCATAA ATCCACCGAAGGATCTAA
atpD	Proton motive force generation	TAAAGTCGGTCTGTTCGGTG CGTGATAGAAGTCGTTACCCTC

### Determination of Growth-Promoting Characteristics of Strain JZ-GX1 in the Presence of Different Salt Concentrations

The siderophile production capacity of strain JZ-GX1 was determined as described in the literature (Schywn and Nielands, 1987); the indole acetic acid production capacity was assessed in accordance with the literature (Glickmann and Dessaux, 1995); and the phosphorus dissolving ability was determined according to the method reported by Li et al. (2012).

### Assessment of Seed Germination

Tomato seeds with similar breakage, size and fullness were selected, surface-sterilized with 10% hydrogen peroxide for 10 min, and washed thoroughly with aseptic distilled water to remove the residual hydrogen peroxide, after which the seeds were air-dried. Two groups were established: the control group (H_2_O and LB) and the experimental group (JZ-GX1). The surface-sterilized seeds were soaked in sterile water and then cultured in LB liquid medium with 1 × 10^7^ cfu/mL JZ-GX1 for 24 h. Subsequently, seeds of the same size were evenly distributed in a Petri dish (20 seeds in each dish) containing 10 mL of 100 mmol/L NaCl solution. Four replicates of each treatment were included. Starting on the 2nd day of observation of the artificial climate box, the number of germinated seeds based on a radicle length of 0.2 cm as the germination standard was counted every day. On the 7th day, 10 plants from each treatment were randomly selected to measure their germination length, root length, and fresh weight and to calculate their germination rate and germination energy (Fu et al., 2017).

Germinationrate(%)=(numberofgerminatedseedsbyday 7/totalnumberoftestseeds)×100.

Germinationenergy(%)=(numberofgerminatedseedsbyday 3/totalnumberoftestseeds)×100.

### Statistical Analysis

The data were analyzed by analysis of variance and Duncan’s multiple comparison using SPSS 17.0, and the standard errors of the mean values were calculated (*P* < 0.05).

## Results

### Salt-Tolerant Growth Characteristics of *R. aquatilis* JZ-GX1

**FIGURE 1:**
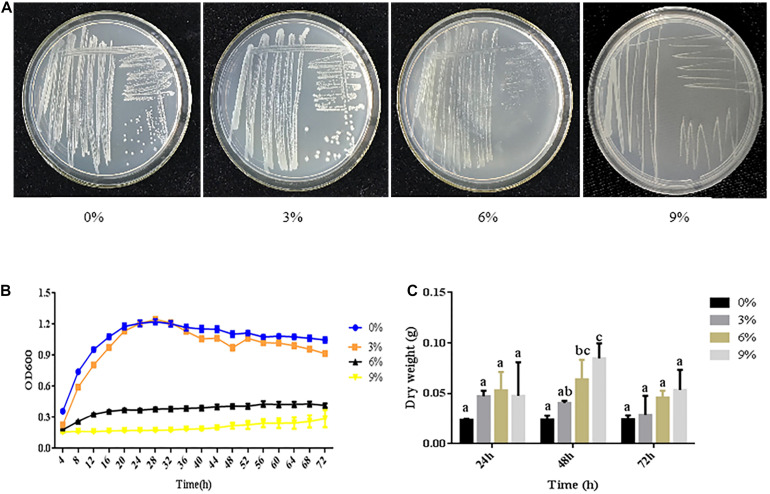
Growth of *R. aquatilis* JZ-GX1 in media with different salt concentrations. **(A)** Images of culture plates. **(B)** Growth curves. **(C)** Cell dry weights. The vertical bars represent the standard deviations of the averages. One-way ANOVA was performed, and Duncan’s *post hoc* test was applied. Different letters indicate significant differences (*P* < 0.05) among the treatments.

The growth of bacteria in media with high salt concentrations is an important indicator of their salt tolerance. *R. aquatilis* JZ-GX1 was able to grow on plates containing 0–9% NaCl, indicating that strain JZ-GX1 is a moderately halophilic bacterium. In the presence of 9% NaCl concentration of, the growth rate was slow, and the colonies were small. Thus, the decrease in the growth rate of strain JZ-GX1 was related to the increase in salt concentration (Figure 1A).

A microplate reader was used to generate growth curves of strain JZ-GX1 cultured in LB medium with different salt concentrations. Strain JZ-GX1 had the shortest lag phase when cultured in the presence of 1%, while the lag phase reached 18 h in the presence of 9% NaCl. After entering the logarithmic phase of growth, the fastest growth rate of strain JZ-GX1 was obtained with a salt concentration of 3%, and the lowest growth rate of strain JZ-GX1 was observed with a salt concentration of 9%. After entering the stationary phase, the maximum OD600 value exceeded 1.0 for strain JZ-GX1 when cultured in the presence of 3% NaCl, with this phase lasting longer when cultured in medium containing 0 and 3% NaCl than 6% NaCl. Increasing the NaCl concentration to 9% significantly prolonged the lag phase, decreased the growth rate at each growth period, and significantly inhibited the growth of *R. aquatilis* JZ-GX1. In addition, the lowest OD value observed after entering the stationary phase was detected at this salt concentration, with a maximal OD value of 0.241. Furthermore, almost no growth in the logarithmic phase was detected at this salt concentration, and the growth was seriously inhibited (Figure 1B). A further increase in the salt concentration reduced the tolerance and survival rate of the bacteria.

In terms of biomass, salt stress (3, 6, and 9%) increased the biomass of strain JZ-GX1 compared to that obtained in the absence of salt stress. At a salt concentration of 0%, no significant difference in the cell dry weight was observed over time. At a salt concentration of 3% NaCl, the dry weight of JZ-GX1 decreased slowly over time. At salt concentrations of 6 and 9%, the dry weight of strain JZ-GX1 first increased and then decreased over time (Figure 1C).

### Electron Microscopy Observations of *R. aquatilis* JZ-GX1 in the Presence of Different Salt Concentrations and Secretion of Extracellular Polysaccharides

**FIGURE 2:**
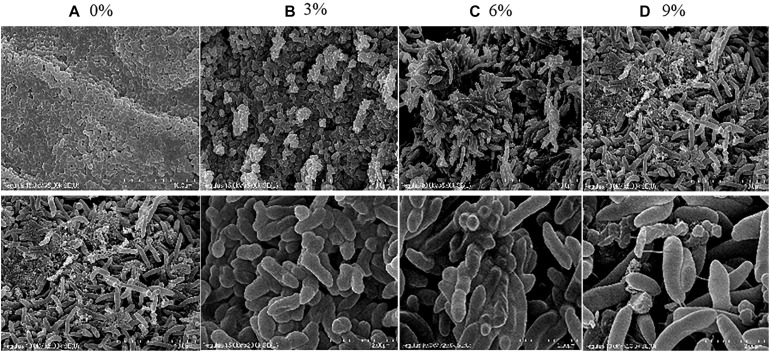
Morphological changes in *R. aquatilis* JZ-GX1 at different salinities. **(A–D)** show scanning electron microscopy images of cells in the presence of 0, 3, 6, and 9% NaCl, respectively, with the scale bars indicating 10 or 2 μm.

As shown in Figure 2, the cell morphology of *R. aquatilis* JZ-GX1 was altered under different levels of salt stress. In the presence of 0% NaCl, the cells were relatively short and regular in shape and presented many visible folds on the surface. In the presence of 3% NaCl, the bacteria became slightly longer and showed a sparse arrangement, and the visible folds on the cell surface were decreased and relatively smooth. In medium containing 6% NaCl, the bacteria adsorbed and adhered together, presumably due to the secretion of EPSs by the cells. In the presence of 9% NaCl, the bacteria were notably longer and arranged in a disorderly manner, cell fragments were detected, the cell morphology was damaged, and breakage was observed during the cell division. In general, increases in the degree of salt stress were associated with increases in cell length. The length and width of the cells treated with 9% salt were 5.12- and 1.41-fold higher than those observed with 0% salt, respectively. The results showed that strain JZ-GX1 can resist a high salt environment by altering its cell morphology.

**FIGURE 3:**
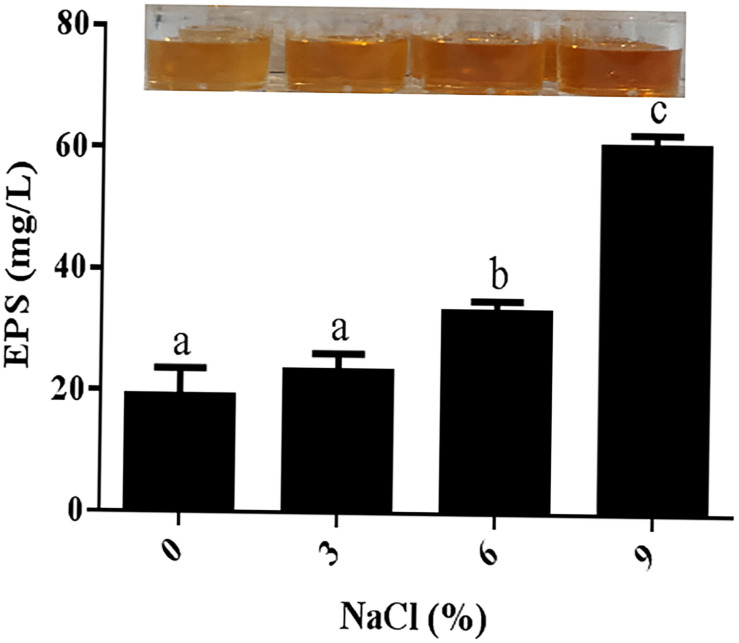
Determination of extracellular polysaccharide levels produced by *R. aquatilis* JZ-GX1 at different salt concentrations. The vertical bars represent the standard deviations of the averages. One-way ANOVA was performed, and Duncan’s *post hoc* test was applied. Different letters indicate significant differences (*P* < 0.05) among the treatments.

The production of EPSs by many halotolerant bacteria is a growth strategy that can promote the formation of a protective layer around cells under adverse conditions to protect cells from the external environment. Therefore, the potential role of EPSs in the salt tolerance of JZ-GX1 was evaluated. As shown in Figure 3, strain JZ-GX1 could secrete EPSs in the presence of different salinities, and the EPS yield increased with increasing salt concentration (0, 3, 6, and 9% NaCl), reaching maximum levels at 9% NaCl (60.6983 mg/L).

### Accumulation of Compatible Solutes by *R. aquatilis* JZ-GX1 in the Presence of Different Salt Concentrations

**FIGURE 4:**
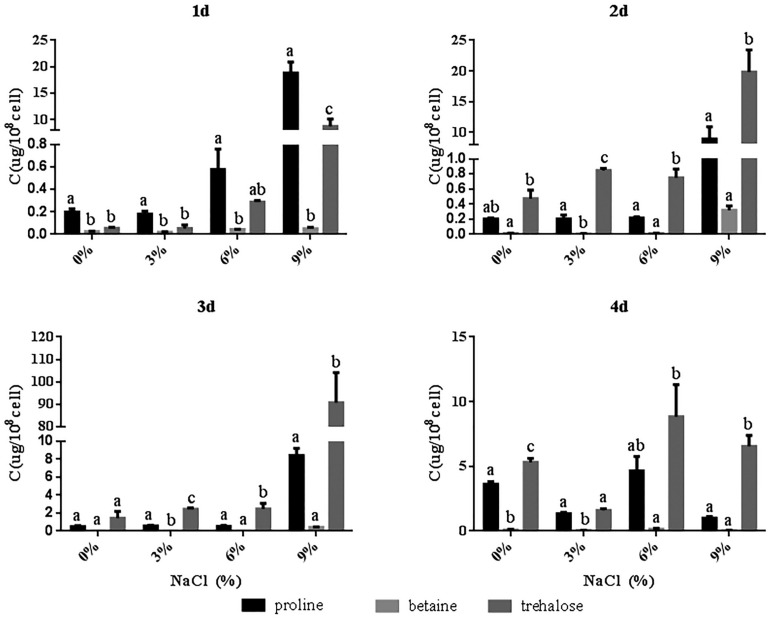
Compatible solutes accumulated by *R. aquatilis* JZ-GX1 in the presence of different salt concentrations. The vertical bars represent the standard deviations of the averages. One-way ANOVA was performed, and Duncan’s *post hoc* test was applied. Different letters indicate significant differences (*P* < 0.05) among the treatments.

To determine the type and content of compatible solutes in the JZ-GX1 strain, the accumulation of proline, betaine and trehalose in the presence of different concentrations of NaCl (0, 3, 6, and 9%) was assessed. As shown in Figure 4, the accumulation of proline and trehalose gradually increased with increases in the NaCl concentration on the 1st day, but no obvious change in the betaine content was detected. The highest accumulation of proline, betaine and trehalose was detected with 9% NaCl on the 1st, 2nd, and 3rd days, respectively, and on the 4th day of fermentation with 6% NaCl. Compared to the results obtained under salt-free conditions, the contents of proline, betaine and trehalose increased by 28.15, 46.55, and 66.63%, respectively. In addition, on the 1st day, strain JZ-GX1 primarily relied on proline to resist salt stress, while trehalose was relied upon at later stage, indicating that were selective in the accumulation of soluble solutes. These results suggest that strain JZ-GX1 may alleviate salt stress by regulating the content of compatible solutes *in vivo*, and the accumulation of trehalose is the primary protective mechanism used by this bacterium against high salt stress.

### Analysis of Na^+^ and K^+^ Concentrations in *R. aquatilis* JZ-GX1 in the Presence of Different Salt Concentrations

**FIGURE 5:**
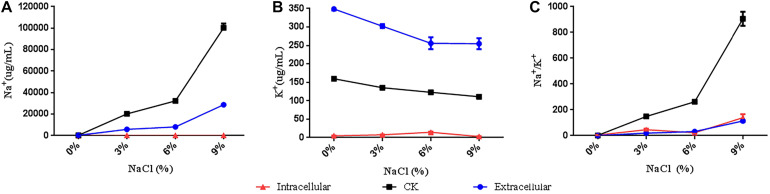
Na^+^ and K^+^ concentrations of *R. aquatilis* JZ-GX1 in the presence of different salt concentrations. **(A)** Na^+^ content. **(B)** K^+^ content. **(C)** Na^+^/K^+^.

*R. aquatilis* JZ-GX1 cells were analyzed by flame photometry, and a large amount of Na^+^ was detected in the intracellular/extracellular spaces (Figure 5). The concentration of Na^+^ in strain JZ-GX1 was lower than that in LB liquid medium (CK), a finding that was observed both intracellularly and extracellularly. Analysis of the accumulation of K^+^ showed that the intracellular K^+^ concentration was lower than that of CK, whereas the extracellular K^+^ concentration was higher than that of CK. Further analysis of the extracellular Na^+^/K^+^ concentrations showed that the intracellular and extracellular Na^+^/K^+^ concentrations of strain JZ-GX1 were lower than those of CK under the four salt concentration gradients. These findings show that *R. aquatilis* JZ-GX1 can accumulate K^+^ and increase the Na^+^/K^+^ ratio to cope with high salt environments, demonstrating another osmotic adaptation mechanism of *R. aquatilis* JZ-GX1.

### Differential Expression of Salt Tolerance-Related Genes in *R. aquatilis* JZ-GX1 in the Presence of Different Salt Concentrations

**FIGURE 6:**
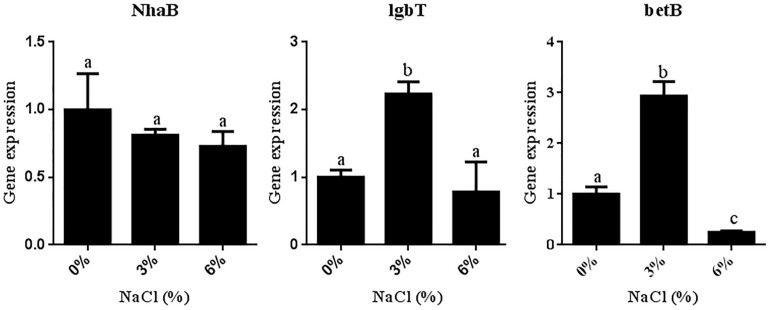
Differential expression of salt tolerance-related genes in *R. aquatilis* JZ-GX1 in the presence of different salt concentrations. The vertical bars represent the standard deviations of the averages. One-way ANOVA was performed, and Duncan’s *post hoc* test was applied. Different letters indicate significant differences (*P* < 0.05) among the treatments.

To further elucidate the molecular mechanism underlying the salt tolerance of JZ-GX1, the target genes NhaB (encoding Na^+^/H^+^ antiporter NhaB), lgbT (encoding L-proline/glycine) and betB (encoding betaine) were selected to analyze the effect of NaCl on the expression of salt tolerance-related genes in strain JZ-GX1 at the transcriptional level. As shown in Figure 6, the level of NhaB transcription decreased with increasing salinity (0, 3, and 6% NaCl). In contrast, whereas the transcriptional levels of lgbT and betB first increased and then decreased with increasing salinity, the maximal values of these genes were obtained with 3% NaCl and reached 2.23- and 2.94-fold higher values than those observed in the absence of NaCl, respectively.

### Effects of Different Salt Concentrations on the Growth-Promoting Characteristics of *R. aquatilis* JZ-GX1

**FIGURE 7:**
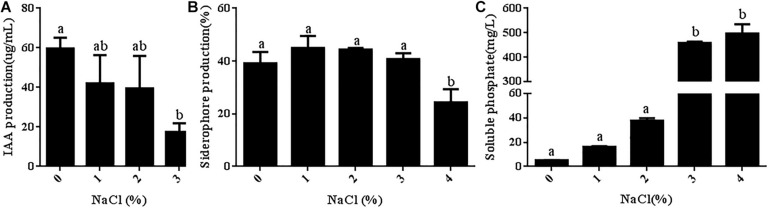
Determination of JZ-GX1-related growth-promoting characteristics in the presence of different salt concentrations. **(A)** IAA content. **(B)** Siderophore content. **(C)** Phosphorus concentration. The vertical bars represent the standard deviations of the averages. One-way ANOVA was performed, and Duncan’s *post hoc* test was applied. Different letters indicate significant differences (*P* < 0.05) among the treatments.

Some PGPRs can promote plant growth under stress by secreting IAA. Therefore, the ability of strain JZ-GX1 to secrete IAA was also assessed in the present study, and the results are shown in Figure 7A. Quantitative analysis showed that strain JZ-GX1 could secrete a high concentration of IAA, even in TSB medium with a salt concentration of 3%. Siderophores are bioactive substances secreted by PGPRs that can increase the absorption of iron in plants and inhibit the growth of pathogens. In the present study, the ability of strain JZ-GX1 to synthesize siderophores was determined. As shown in Figure 7B, the siderophore production of strain JZ-GX1 first increased and then decreased as the salt concentration increased, and the highest content of siderophores (44.93%) was obtained with a salt concentration of 1%. Appropriate salt concentrations could promote the secretion of siderophores by strain JZ-GX1, but the level of secreted siderophores began to decrease when the salt concentration reached 4%. By producing iron carriers, strain JZ-GX1 can help plants obtain iron from soil while controlling plant pathogens. An analysis of the ability of strain JZ-GX1 to dissolve inorganic phosphorus under different salt concentrations showed that increases in the salt concentration significantly improved the phosphorus-solubilizing ability of strain JZ-GX1 (Figure 7C).

### Effect of *R. aquatilis* JZ-GX1 on Tomato Seed Germination Under Salt Stress

**FIGURE 8:**
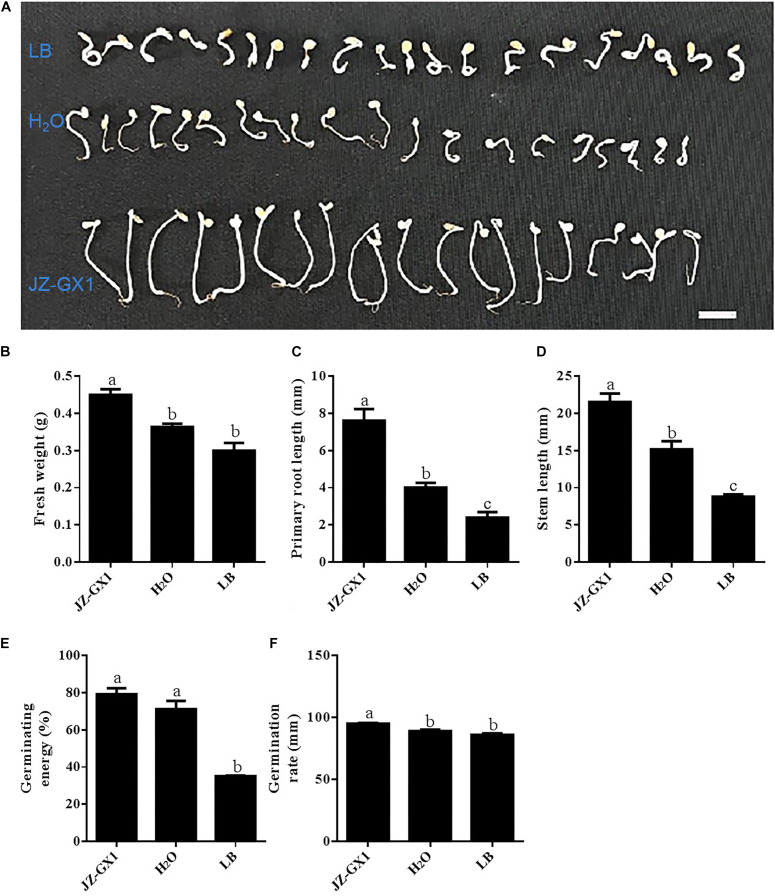
Effects of *R. aquatilis* JZ-GX1 on tomato seed germination and seedling growth under salt stress (100 mM). **(A)** Phenotype. **(B)** Fresh weight. **(C)** Primary root length. **(D)** Stem length. **(E)** Germination energy. **(F)** Germination rate. The vertical bars represent the standard deviations of the averages. One-way ANOVA was performed, and Duncan’s *post hoc* test was applied. Different letters indicate significant differences (*P* < 0.05) among the treatments.

To determine whether strain JZ-GX1 can promote plant growth under salt stress, we used tomato as the target plant (Figure 8A). After being subjected to salt stress for 7 days, the fresh weight of the seedlings treated with strain JZ-GX1 was 23.97 and 50.005% higher than those obtained with the H_2_O and LB treatments, respectively (Figure 8B). In addition, the primary root length of the JZ-GX1 strain-treated seedlings was approximately 1.9- and 3.2-fold longer than those of the seedlings subjected to the H_2_O and LB treatments, respectively (Figure 8C). Furthermore, the stem length of the seedlings treated with strain JZ-GX1 was 41.88 and 144.64% higher than those obtained with the H_2_O and LB treatments, respectively (Figure 8D). As shown in Figures 8E,F, treatment with strain JZ-GX1 significantly increased the germination rate and germination energy of tomato seedlings under salt stress. The results showed that the germination percentage and germination potential of tomato seeds treated with strain JZ-GX1 were significantly higher than those of the seeds treated with H_2_O and LB, and the fresh weight, primary root length and stem length of the tomato seedlings were significantly increased by treatment with strain JZ-GX1.

## Discussion

Salinity is an abiotic stress in nature that exerts harmful effects on organisms. When cells are exposed to high-salt conditions, osmotic pressure pulls unbound water molecules out of the cells to yield a highly concentrated cytoplasm, which impairs cell function and can lead to cell death. Therefore, organisms living in high-salt environments need to have specific adaptation mechanisms (Weinisch et al., 2018). As cells adapt to a new osmotic environment, their biomass will change. In the present study, salt stress (3, 6, and 9%) increased the biomass of strain JZ-GX1 compared to that observed under non-salt stress conditions. The highest biomass of strain JZ-GX1 was obtained after 48 and 72 h of exposure to 9% NaCl, and the dry weight of strain JZ-GX1 at high salt concentrations was higher than that observed at low salt concentrations. Some studies have shown that EPSs can protect PGPRs from external stress during inoculation (Arora et al., 2020). According to relevant studies, the EPS yield shows a positive correlation with salt concentrations of 0–6% NaCl, reaching a maximum value (222.67 mg/g) at 6% NaCl. In the presence of higher NaCl concentrations (8–10%), the EPSs yield was shown to gradually decrease (Qiu et al., 2020). Khanna (2015) also observed similar results. In the present study, the EPS content produced by strain JZ-GX1 increased as the salt concentration increased, an effect was observed up to a salt concentration of 9%, which promoted the formation of a biofilm barrier. These findings indicated that one of the strategies used by strain JZ-GX1 to limit salt absorption involves reducing the intake of sodium by capturing sodium ions in its EPS matrix.

Previous studies have shown that halophilic bacteria under high salinity need to change their morphology to adapt to salt stress (Kogej et al., 2006). The phenomenon of early bacterial cell lengthening has been observed in *Pseudomonas* sp., but in *Rhodopseudomonas palustris*, this process is limited to the metabolism of selenium (Manter, 2009; Li et al., 2014). Mccready et al. (1966) suggested that the changes in growth of bacterial cells treated with selenium is due to the inhibition of cell division. The results of the present study are consistent with previously results describing significant lengthening of PGPR cells under abiotic stress (Yanyun et al., 2018). To date, it remains unclear why bacteria have evolved to produce larger cells under stressful conditions, but it might be because when bacteria encounter harmful substances in the environment (such as heavy metals, active oxygen species, drugs, etc.), mother cells can store more nutrients needed for the life activities of future generations by increasing the cell surface area (Philippe et al., 2009). Interestingly, although strain JZ-GX1 cells were damaged to different degrees under different salt concentrations, they could still grow normally.

In a high salt environment, the establishment of intracellular ion homeostasis is important for the survival and growth of microorganisms (Plemenitas et al., 2016). Another strategy for avoiding high salt concentrations in the cytoplasm is to pump ions out of the cell. Na^+^/H^+^ antiporters, such as NhaA, an antiporter of *Escherichia coli* and many bacteria (Hunte et al., 2005), aid in pumping out excess sodium ions (Ruppel et al., 2013). In prokaryotes, Na^+^/H^+^ antiporters play an important role in salt tolerance. The results of the present study revealed that the expression of NhaB in *R. aquatilis* JZ-GX1 decreased with as the salt concentration increased, which could effectively reduce the continuous intake of sodium ions. In addition to NhaB, *R. aquatilis* JZ-GX1 can also resist high osmotic pressure by altering the expression of other salt tolerance-related genes. It is worth noting that Na^+^ not only participates in the regulation of osmotic pressure in the form of ions but also plays an important role in the regulation of the osmotic equilibrium of organically compatible solutes as some important substances for the transport of amino acids. Some microorganisms also produce organic osmotic substances that accumulate in the cytoplasm and resist osmotic pressure under high salt stress. To adapt to the balance of intracellular osmotic pressure, some osmotic regulators and protective substances, such as betaine (Wu et al., 2018) and proline (Nadeem et al., 2007), need to be synthesized and accumulate. Some moderately halophilic bacteria often exhibit selectivity for the accumulation of compatible solutes, reflecting the selective accumulation of several specific compatible solutes at different growth periods and in the presence of different salt concentrations. For example, when *Halomonas israelensis* was cultured at salinities lower than 3.5%, the major compatible solute that accumulated was trehalose, whereas the primary compatible solute that accumulated during cultivation at high salinities was tetrahydropyrimidine (He et al., 2005). *Bacillus* sp. I121 primarily accumulates proline under high salt concentrations and tetrahydropyrimidine at higher environmental salt concentrations, primarily maintaining its cellular osmotic pressure by accumulating tetrahydropyrimidine as a compatible solute (Jiang et al., 2010). In the present study, an analysis of the accumulation of three common compatible solutes (proline, betaine and trehalose) by *R. aquatilis* JZ-GX1 in the presence of different salt concentrations revealed that this strain primarily accumulated trehalose in the presence of high environmental salt concentrations, but no obvious trend was detected at low salt concentrations. In addition, the storage of osmotic substances represents a successful stress response mechanism that promotes the ability of bacteria to limit their water loss and increase the concentration of potassium ions in their cytoplasm. These results indicate that *R. aquatilis* JZ-GX1 adopts the “salting-out” strategy (the process of removing sodium ions and accumulating compatible solutes) to address high osmotic damage.

Microorganisms inhabiting plant roots form a complex ecological community with plants, affecting plant growth and productivity through their metabolic activities (Baek et al., 2010). The interactions between plants and beneficial microorganisms such as PGPRs can alleviate abiotic stress and increase the tolerance of plants to adverse growth conditions (Rodriguez et al., 2008; Aghili et al., 2014). In addition, PGPRs can also improve plant growth through indirect and direct mechanisms, including atmospheric nitrogen fixation, inorganic phosphorus hydrolysis, iron chelation, antibiotics, volatile compound production, hydrolase synthesis or plant hormone production (Babalola, 2010). A quantitative analysis confirmed that *R. aquatilis* JZ-GX1 can produce IAA and siderophores and has the ability to dissolve phosphorus under salt stress. Our results are consistent with those reported by Fatima et al. (2020). *Alcaligenes* AF7 has the ability to dissolve phosphorus under salt stress, and this ability promotes the ability of plants to grow under salt stress. Seed germination and early seedling growth are typically the most sensitive stages affected by salinity (Foolad, 2004), and mitigating the effects of salt at these early stages will increase the probability of successful crop growth under salt stress (Ashraf et al., 2003). In the present study, JZ-GX1 promoted the germination rate of tomato seeds under salt stress and enhanced the tolerance of tomato to salt stress by promoting shoot and root development and photosynthesis. This finding was consistent with those reported by Nadeem et al. (2013). Compared to the untreated control, seedings in natural saline land inoculated with *Pseudomonas putida*, *Enterobacter cloacae*, *Serratia ficaria*, and *Pseudomonas fluorescens* significantly increased the germination rate and germination index of wheat seeds and significantly increased wheat yields in saline-alkali land (Nadeem et al., 2013). The results of the present study demonstrated that strain JZ-GX1 has good application prospects for promoting plant growth under salt stress.

PGPR have value in a wide range of applications by inducing plants to resist adverse environmental conditions. We will perform pot experiments in the future to assess how the PGPR JZ-GX1 can improve early tomato seedling growth, including by studying its metabolic regulatory processes and associated molecular mechanisms in depth.

## Data Availability Statement

The raw data supporting the conclusions of this article will be made available by the authors, without undue reservation.

## Author Contributions

P-SL completed the data analysis and the first draft of the manuscript. P-SL and W-LK completed the experiments. X-QW directed the experimental design, data analysis, and manuscript writing and revision. All the authors read and agreed on the final text.

## Conflict of Interest

The authors declare that the research was conducted in the absence of any commercial or financial relationships that could be construed as a potential conflict of interest.
